# Chlorogenic Acid Attenuates Dextran Sodium Sulfate-Induced Ulcerative Colitis in Mice through MAPK/ERK/JNK Pathway

**DOI:** 10.1155/2019/6769789

**Published:** 2019-04-18

**Authors:** Wenyan Gao, Changhong Wang, Li Yu, Tianjiao Sheng, Zhuolin Wu, Xiaoqian Wang, Dongqi Zhang, Yifan Lin, Yang Gong

**Affiliations:** Department of Traditional Chinese Medicine, The General Hospital of Northern Theater Command, No. 83 Wenhua Road, Shenyang 110016, China

## Abstract

**Objective:**

Observe the protective effect of chlorogenic acid on dextran sulfate-induced ulcerative colitis in mice and explore the regulation of MAPK/ERK/JNK signaling pathway.

**Methods:**

Seventy C57BL/6 mice (half males and half females) were randomly divided into 7 groups, 10 in each group: control group (CON group), UC model group (UC group), and sulfasalazine-positive control group (SASP group), chlorogenic acid low dose group (CGA-L group), chlorogenic acid medium dose group (CGA-M group), chlorogenic acid high dose group (CGA-H group), and ERK inhibitor + chlorogenic acid group (E+CGA group). The effects of chlorogenic acid on UC were evaluated by colon mucosa damage index (CMDI), HE staining, immunohistochemistry, ELISA, and Western blot. The relationship between chlorogenic acid and MAPK/ERK/JNK signaling pathway was explored by adding ERK inhibitor.

**Results:**

The UC models were established successfully by drinking DSS water. Chlorogenic acid reduces DSS-induced colonic mucosal damage, inhibits DSS-induced inflammation, oxidative stress, and apoptosis in colon, and reduces ERK1/2, p -ERK, p38, p-p38, JNK, and p-JNK protein expression. ERK inhibitor U0126 reversed the protective effect of chlorogenic acid on colon tissue.

**Conclusion:**

Chlorogenic acid can alleviate DSS-induced ulcerative colitis in mice, which can significantly reduce tissue inflammation and apoptosis, and its mechanism is related to the MAPK/ERK/JNK signaling pathway.

## 1. Introduction

Ulcerative colitis (UC) is a refractory inflammatory disease of the large intestine [1], and the pathogenesis of UC is not fully understood. In addition to conventional treatment with corticosteroids and amino salicylic acid [2], the treatments of UC are including many new drugs such as TNF-antibody clinically widely used [3–6]. However, patients with ulcerative colitis often relapse after a certain period of treatment, which leads to serious economic and mental problems [7]. In-depth understanding of the pathogenesis and finding effective drug of UC is a hot topic of current research, and it is one of the medical problems urgently needed to be resolved.

**Figure 1:**
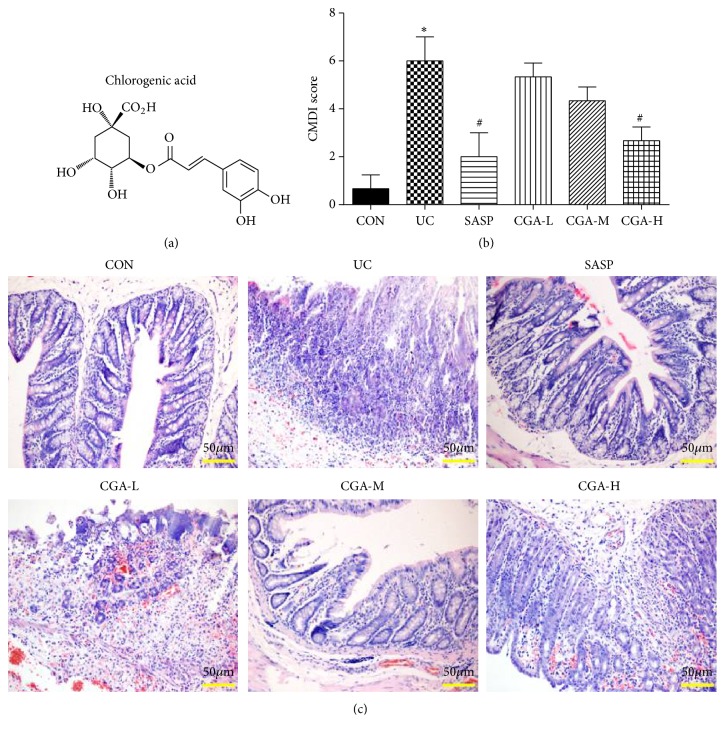
Chlorogenic acid reduces DSS-induced colonic mucosal damage. (a) The chemical construction of chlorogenic acid; (b) CMDI scores were used to assess the degree of intestinal mucosal injury of mice in each group; (c) HE staining (scale bar=50 *μ*m) was used to assess the degree of intestinal mucosal injury of mice in each group; *∗* indicates P<0.05.

Chlorogenic acid: [1,3,4,5-tetrahydroxycyclohexanecarboxylic acid-(3,4-dihydroxycinnamate, CGA)], chemical name 3-O-coffeic with quinic acid, molecular formula C_6_H_18_O_9_, and molecular weight 354.3 (Figure 1(a)) [8]. Chlorogenic acid is mainly found in traditional Chinese medicine honeysuckle, hawthorn, eucommia, and chrysanthemum, especially in coffee [9]. Studies have shown that chlorogenic acid has antioxidation, anti-inflammatory, and antibacterial effects, is free radical scavenging, and improves immune regulation [10–14]. Chlorogenic acid was shown to have a certain therapeutic effect on ulcerative colitis, inhibiting intestinal inflammation, weight loss, diarrhea, and colon shortening, and improving the immune regulation of intestinal microbes [15, 16]. However, the mechanism of chlorogenic acid has not been fully explored.

The pathway of mitogen-activated protein kinase (MAPK) is an important pathway for intracellular signal transduction, which widely presents in eukaryotic cells and is involved in the regulation of cell differentiation, proliferation, division, and apoptosis [17]. Among the MAPK family members, the earliest confirmed pathway is the transduction pathway, which is divided into extracellular signal-regulated kinase 1 (ERK1) and ERK2 [18]. The ERK pathway is a classical pathway for MAPK signaling [19]. In addition to ERK1/2, the MAPK family has another two members, c-Jun N-terminal kinase (JNK) and p38 kinase, which were reported to play a major role in promoting apoptosis [20]. The MAPK signaling pathway is a signaling pathway closely related to the pathogenesis of ulcerative colitis and its activation is considered to be one of the major factors leading to the release of cytokines and inflammatory mediators in ulcerative colitis [21–23]. It has been reported that chlorogenic acid has the regulation on MAPK signaling pathway in liver injury model [24], but the relationship of chlorogenic acid with MAPK signaling pathway in ulcerative colitis model has not been reported. Therefore, this article used the mouse model of ulcerative colitis to observe the protective effect of chlorogenic acid and to explore the regulation mechanism of MAPK / ERK / JNK signaling pathway in the process.

## 2. Materials and Methods

### 2.1. Laboratory Animals and Grouping

70 SPF C57BL/6 adult mice, half male and half female, weighing 25±3g, were provided by the Laboratory Animals Department of Shenyang Military Region General Hospital (rodent use license: SYXK(Jun)20120007; rodent production license: SCXK (Jun) 20120006); animal experiment was approved by the Experimental Animal Ethics Committee of General Hospital Shenyang Military Region (NO2017011).

The mice were randomly divided into 7 groups, 10 in each group: control group (CON group), UC model group (UC group), sulfasalazine positive control group (SASP group), chlorogenic acid low dose group (CGA-L group), chlorogenic acid middle dose group (CGA-M group), chlorogenic acid high dose group (CGA-H group), and ERK inhibitor plus chlorogenic acid treatment group (E+CGA group). In addition to the CON group, the ulcerative colitis model was made as described in the literature [25]. The dextran sodium sulfate (DSS) was dissolved in distilled water to prepare a 5% DSS solution, and the mice were allowed to freely drink the solution for 7 days, and then to drink distilled water for 14 days. After preparation of UC model, mice in the SASP group were given 100 mg/kg/day SASP through intragastrical administration for 10 days. In the CGA low, medium, and high dose groups mice were given 30, 60, and 120 mg/kg/day chlorogenic acid through intragastrical administration for 10 days. In the E+CGA group, U0126 (0.2 mg/kg), the inhibitor of ERK, was injected into the tail vein.

The disease activity index was used daily to observe the mice. Mice were anesthetized with pentobarbital sodium and the abdominal cavity was opened. After the blood was collected through the abdominal aorta, the anus was taken to the colon of the ileocecal section, and the mesenteric longitudinal section was taken, expanded, and fixed. The gross morphological changes were observed under the anatomical microscope. Each animal takes one tissue specimen in the distal colon, transverse colon, and ascending colon and at least one tissue in the severe inflammation or ulcer. These tissue samples were half fixed in 4% paraformaldehyde and half stored in the liquid nitrogen.

### 2.2. Colon Mucosal Damage Index (CMDI)

According to the methods in the literature, CMDI evaluation was briefly as follows: (1) ulcer and inflammation: 0 points, normal; 1 point, focal congestion, no ulcer; 2 points, ulcer without congestion or thickening of the intestinal wall; 3 points, one place with inflammatory ulcer; 4 points, ulcers and inflammatory sites in two places; 5 points, the main part of the damage along the colon extension ≥1cm; 6-10 points, injury along the length of the colon extended ≥2cm; for each increase of 1 cm damage, the score increased by 1 point; (2) The presence of adhesion: 0 points, normal; 1 point, slight adhesion; 2 points, the main adhesion [26].

### 2.3. HE Staining

Tissue samples fixed in paraformaldehyde were placed in 70%, 80%, 90%, 95%, and 100% different concentrations of alcohol, and xylene was transparent, dipped in wax, embedded in wax blocks, and sliced 4 *μ*m each slice, and then dewaxed, hematoxylin stained for 5 minutes, washed by PBS, differentiated in 1% hydrochloric acid alcohol, dyed in eosin solution for 30 seconds, dehydrated in gradient alcohol, was in transparent treatment, neutral gum sealed, and observed in light microscope for pathological changes.

### 2.4. Immunohistochemistry

The sections of colon tissue were deparaffinized by xylene, hydrated with gradient ethanol, incubated with 0.1% Tritonx-100 for 30 min, and rinsed 3 times with PBS for 5 min each. The sections were blocked by 5% BSA and 10% sheep serum successively for 30 min. The sections were incubated separately with primary antibody ERK1/2 (ab17942, Abcam), p-ERK (ab192591, Abcam), p38 (ab31828, Abcam), p-p38 (ab47363, Abcam), JNK (ab208035, Abcam), and p-JNK (ab124956, Abcam) in a wet box at 4°C overnight, washed 3 times with PBS for 5 min per time and second antibody at 37°C for 40 minutes, and washed with PBS 3 times for 5 minutes per time. Horseradish peroxidase-labeled streptavidin protein was added dropwise at 37°C for 40 minutes. The sections were rinsed 3 times with PBS for 5 minutes each time, developed by DAB, microscopically observed, and photographed.

### 2.5. ELISA Essay

Enzyme-linked immunosorbent assay (ELISA) kit (Cloud-Clone Corp., USA) was used to detect the expression of TNF-*α* (SCA133Mu, USCN), IL-1*β* (CSB-E08054m, CUSABIO), IL-6 (SEA079Mu, USCN), IL-10 (SEA056Mu, USCN), PAF (CEA526Ge, USCN), PGE2 (CEA538Ge, USCN), MPO (SEA601Mu, USCN), and SOD (SES134Mu, USCN) in serum and tissues of mice. The operation steps are carried out according to the kit operating instructions: after the kit is equilibrated to room temperature (20 ~ 25°C), the corresponding reaction plate wells were added 100 ul standard and 100 ul diluted sample, then mixed and incubated for 20 min at room temperature, then washed with washing buffer, adding 100 ul serum sample to each well, and incubated at 37°C for 2 h; after washing, l00ul HRP labeled secondary antibody per well was added and the plates were incubated for 30 min at 37°C and washed, and then 50 ul solution A and 50 ul solution B were added, and there was coloration for 15 min in the dark, adding 50 ul of the stop solution. The OD value was measured at 450 nm using a microplate reader (Bio-rad 680), and the concentration was calculated by a standard curve.

### 2.6. Western Blot

The protein sample was added to the corresponding proportion of SDS gel loading buffer and boiled for 5 min. After SDS-PAGE electrophoresis and transferred membrane, the membrane was blocked 5% skim milk in PBST buffer, at room temperature for 1 hour and washed with PBST for 3 times. Then ERK1/2, p-ERK, p38, p-p38, JNK, p-JNK, and Bcl-2 (ab321124, Abcam), Bax (ab53154, Abcam), and Caspase3 (ab44976, Abcam) (1:500) primary antibody were added, and the membranes were incubated overnight at 4°C, washed 3 times with PBST, and incubated in the corresponding secondary antibody labeled with horse. Then the membranes were added by radish peroxidase and incubated for 1 hour at room temperature. The membrane was washed 3 times and ECL developed. The gel imaging system was photographed, and the image J software was used to analyze the gray value.

### 2.7. Statistical Analysis

Statistical analysis was performed using SPSS 20.0 software, and the measurement data were analyzed by t-test and analysis of variance. Data represent mean ± standard deviation, and all statistical tests are two-sided probability tests. P < 0.05 difference was statistically significant.

## 3. Results

### 3.1. Chlorogenic Acid Reduces DSS-Induced Colonic Mucosal Damage

DSS-induced mouse ulcerative colitis model was administrated with SASP or various doses of chlorogenic acid; the CMDI score and HE staining were taken to evaluate the degree of intestinal mucosal damage. Compared with the CON group, the CMDI scores of the UC group were significantly higher (P<0.05). Compared with the UC group, the SASP group and the CGA-H group could significantly reduce the CMDI score of the mice (P<0.05) (Figure 1(b)), but there was no significant difference between CGA-L, CGA-M groups and UC group; HE staining was used to observe the pathological changes of intestinal tissue in each group. Compared with the CON group, the UC group showed pathological changes such as mucosal epithelial cell degeneration, necrosis, and inflammatory cell infiltration, the CGA-L and CGA-M groups are similar to the UC group, mild epithelial degeneration/necrosis and a small number of inflammatory cells could be observed in the CON group, SASP group, and CGA-H group (Figure 1(c)).

### 3.2. Chlorogenic Acid Inhibits DSS-Induced Colonic Mucosal Inflammation

**Figure 2:**
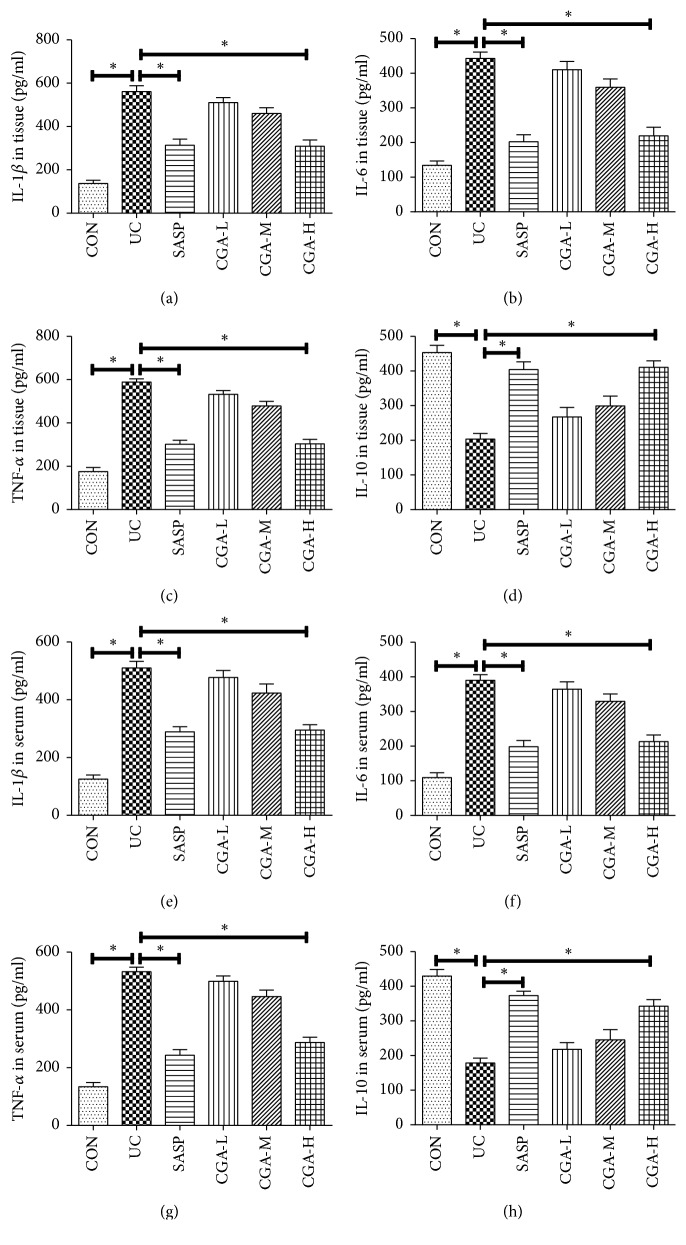
Chlorogenic acid inhibits DSS-induced colonic mucosal inflammation. ELISA assay was used to detected inflammatory factors in the intestinal tissues and serum. (a) IL-1*β*, (b) IL-6, (c) TNF-*α*, and (d) IL-10 level in the intestinal tissues; (e) IL-1*β*, (f) IL-6, (g) TNF-*α*, and (h) IL-10 level in the serum; *∗* indicates P<0.05.

The ELISA essay was used to detect the changes of inflammatory factors in mice and intestinal tissues (Figure 2). Compared with CON, the expression levels of inflammatory factors IL-1*β*, IL-6, and TNF-*α* in serum and colon tissues of UC group were significantly increased. The expression of IL-10 was significantly decreased (P<0.05). Compared with UC group, the levels of IL-1*β*, IL-6, and TNF-*α* in CGA-H group were significantly decreased, and the expression of IL-10 was significantly increased (P<0.05). There was no significant difference in the SASP group compared with the CGA-H group (P>0.05). It was suggested that chlorogenic acid could inhibit the inflammatory reaction of mucosal tissue induced by DSS in ulcerative colitis, but there was no significant difference between CGA-L, CGA-M groups and UC group (P>0.05).

### 3.3. Chlorogenic Acid Improves DSS-Induced Oxidative Stress in Colon

**Figure 3:**
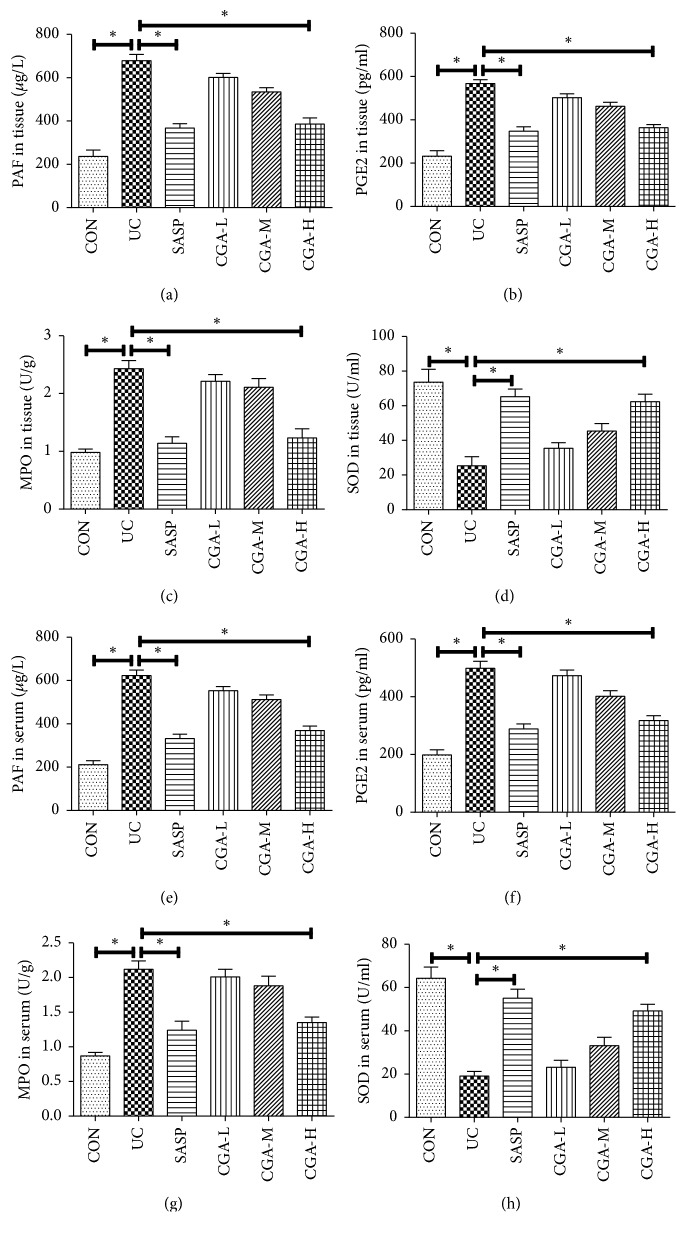
Chlorogenic acid improves DSS-induced oxidative stress in colon. ELISA assay was used to detect the changes of oxidative stress-related factors in mice intestinal tissues and serum. (a) PAF, (b) PGE2, (c) MPO, and (d) SOD level in the intestinal tissues; (e) PAF, (f) PGE2, (g) MPO, and (h) SOD level in the serum; *∗* indicates P<0.05.

The changes of oxidative stress-related factors in blood and intestinal tissues were detected by ELISA essay (Figure 3). Compared with CON group, the PAF, PGE2, and MPO were upregulated in serum and colon tissue of UC group and the SOD was significantly downregulated (P<0.05). Compared with the CGA-H group, there was no significant difference in the SASP group (P>0.05). Compared with UC group, there was no significant difference in CGA-L and CGA-M (P>0.05). Therefore, the CGA-H group was selected for subsequent experiments. The above results suggested that the antioxidant substances in the rats after ulcerative colitis are reduced and the oxidative damage is aggravated and chlorogenic acid can effectively improve the damage.

### 3.4. Chlorogenic Acid Alleviates DSS-Induced Colonic Mucosal Apoptosis

**Figure 4:**
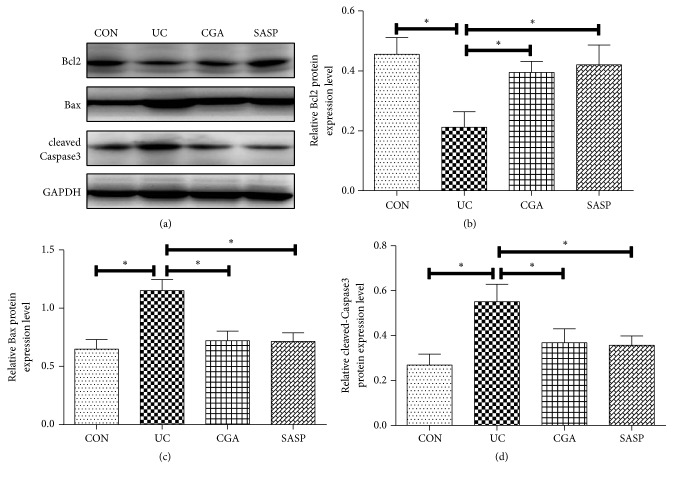
Chlorogenic acid alleviates DSS-induced colonic mucosal apoptosis. Western blot assay was performed to detect the expression levels of (a, b) Bcl-2, (a, c), and (a, d) cleaved-Caspase3; *∗* indicates P<0.05.

To observe the apoptosis of colon tissue, Western blot confirmed that compared with the CON group, the expression of Bcl-2 protein was significantly decreased and the expression of Bax and Caspase3 protein was significantly increased in the DSS-induced model group (P<0.05). In contrast, Bcl-2 protein expression was increased in the CGA group and the SASP group and Bax and cleaved-Caspase3 protein expression were decreased (P<0.05) (Figures 4(a)-4(d)). This indicates that chlorogenic acid can significantly inhibit the apoptosis of intestinal tissue cells induced by DSS in ulcerative colitis.

### 3.5. Effect of Chlorogenic Acid on the Expression of MAPK/ERK/ JNK Pathway-Related Proteins in DSS Mice

**Figure 5:**
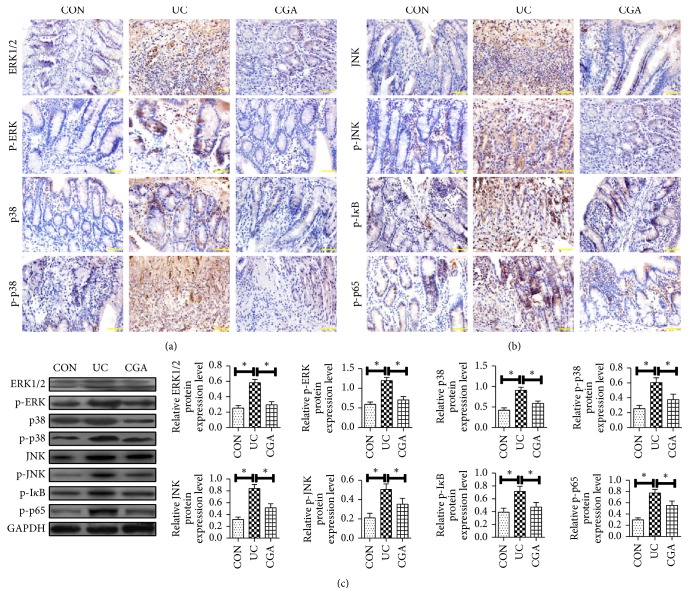
Effect of chlorogenic acid on the expression of MAPK/ERK/ JNK pathway-related proteins in DSS mice. The expression levels of MAPK/ERK/NF-*κ*B pathway-related proteins were detected by IHC and Western blot. IHC assay was performed to detect (a) ERK1/2, p-ERK, p38, p-p38, (b) JNK, p-JNK, p-I*κ*B, and p-p65 of mice in each group (scale bar=50 *μ*m); (c) Western blot assay was performed to detect the expression levels of ERK1/2, p-ERK, p38, p -p38, JNK, p-JNK, p-I*κ*B, and p-p65 protein of mice in each group; *∗* indicates P<0.05.

To further investigate the mechanism of action of chlorogenic acid on DSS rats, immunohistochemistry (IHC) (Figures 5(a)-5(b)) and Western blot (Figure 5(c)) were used to detect the expression of MAPK/ERK/JNK pathway-related proteins. The results showed that compared with CON, UC group mucosal tissue ERK1/2, p -ERK, p38, p-p38, JNK, p-JNK, p-I*κ*B, and p-p65 expression increased significantly (P<0.05). Compared with UC group, ERK1/2, p-ERK, p38, p-p38, JNK, p-JNK, p-I*κ*B, and p-p65 in CGA group were significantly reduced (P < 0.05). From this result, it can be seen that the protective effect of chlorogenic acid on DSS-induced ulcerative colitis intestinal tissue is related to the MAPK/ERK/JNK pathway.

### 3.6. Chlorogenic Acid Improves DSS-Induced Ulcerative Colitis in Mice through MAPK/ERK/JNK Signaling Pathway

To further verify that the therapeutic effect of chlorogenic acid on UC is achieved by inhibiting the MAPK/ERK/JNK signaling pathway, the ERK inhibitor U0126 was first administered in mice to block the pathway before DSS-induction. We found that the effects of chlorogenic acid including reduction of the CMDI score and inhibition of intestinal mucosal damage were blocked.

**Figure 6:**
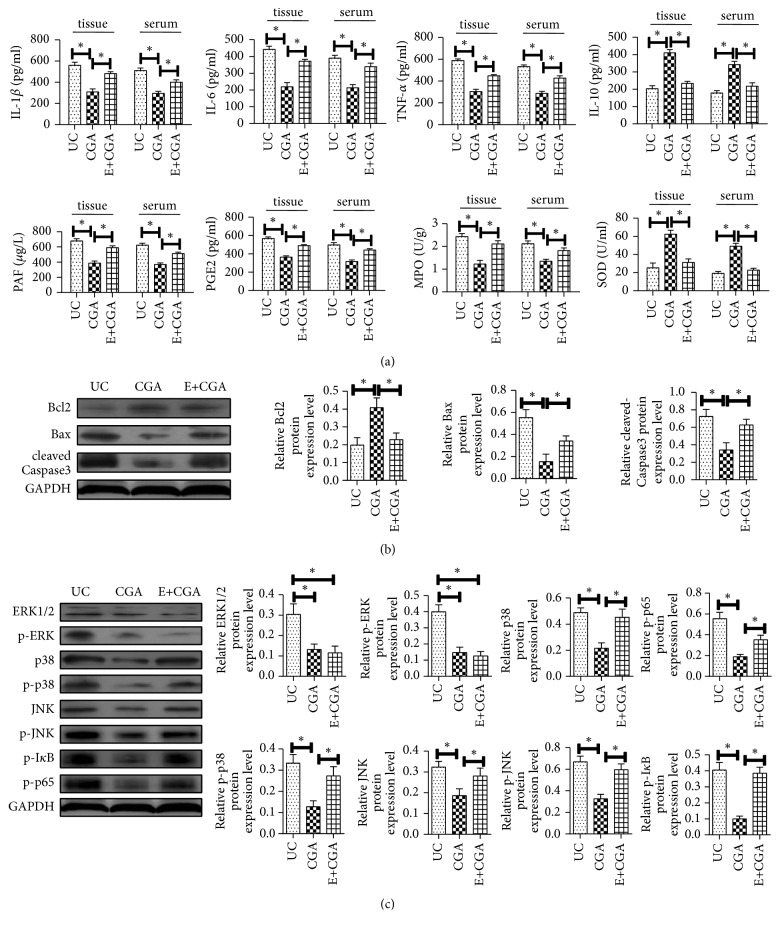
Chlorogenic acid improves DSS-induced ulcerative colitis in mice through MAPK/ERK/JNK signaling pathway. (a) ELISA assays were used to detected IL-1*β*, IL-6, TNF-*α*, IL-10, PAF, PGE2, MPO, and SOD expression levels of mice in each group; (b) Western blot assay was performed to detect the expression levels of Bax, Bcl-2, and cleaved-Caspase3 protein of mice in each group; (c) Western blot assay was performed to detect the expression levels of ERK1/2, p-ERK, p38, p -p38, JNK, p-JNK, p-I*κ*B, and p-p65 protein of mice in each group; *∗* indicates P<0.05.

Meanwhile, chlorogenic acid inhibited inflammatory factors IL-1*β*, IL-6, TNF-*α*, PAF, and PGE2 in serum and colon tissues of mice. The expression of MPO and the promotion of IL-10 expression were all inhibited (Figure 6(a)). In addition, the antiapoptotic effects of chlorogenic acid disappeared after the MAPK/ERK/JNK signaling pathway was blocked (Figure 6(b)). The above results indicated that the signaling pathway was blocked and the therapeutic effect of chlorogenic acid on ulcerative colitis mice was inhibited. In addition, we examined the expression of MAPK/ERK/JNK pathway-associated proteins, and ERK1/2, p38, JNK, IkappaB (I*κ*B), and p65 protein phosphorylation were significant after administration of ERK inhibitors. The decrease indicated that the MAPK/ERK/JNK pathway was blocked and there was no change in the expression of each protein after chlorogenic acid administration, indicating that the effect of chlorogenic acid on MAPK/ERK/JNK pathway disappeared (P<0.05) (Figure 6(c)). These results indicated that chlorogenic acid can alleviate the inflammatory response of ulcerative colitis tissue and inhibition of apoptosis may be achieved through the MAPK/ERK/JNK signaling pathway.

## 4. Discussion

In this study, after successfully establishing a mouse model of ulcerative colitis (UC), we identified that chlorogenic acid can reduce the degree of UC lesions, reduce inflammatory response and apoptosis, play a protective role in DSS-induced UC, and improve the expression of MAPK pathway-associated protein in colonic mucosa induced by DSS. These results suggested its mechanism may be achieved through the MAPK/ERK/JNK pathway.

Ulcerative colitis is a disease of unknown etiology, which is deleterious in the rectum and colon. The clinical features such as diarrhea, sputum, pus and bloody stools, abdominal pain, and ulcerative colitis are recurrent chronic diseases and can occur at any age, for both male and female, more common in young and middle-aged, mostly in western developed countries [27]. In recent years, the incidence of UC is on the rise and tends to be younger [28], but its etiology and pathogenesis have not yet been fully clarified. It is currently believed to be mainly related to genetics, infection, environment, and immunity [29]. Modern medical treatment of UC is mainly based on drugs such as hormones, sulfonamides, and immunosuppressive agents, which can control its symptoms, but the problems of hormone resistance, side effects, and easy repetition are hard to be resolved [30]. In recent years, traditional Chinese medicine ingredients have played an important role in the treatment of ulcerative colitis [31–34]. Studies have shown that hesperidin (flavonoids) may downregulate IL-6 levels in serum by lowering MPO activity and MDA levels in colon tissues [35]. Allicin can regulate CD68, MPO, MDA, and inflammation factor expression to attenuate DSS-induced ulcerative colitis, and its mechanism may be related to the regulation of signal transducer and activator of transcription 3 (STAT3) and nuclear factor-*κ*B (NF-*κ*B) signaling pathway activity [36]. It has recently been found that chlorogenic acid can also have a certain therapeutic effect on ulcerative colitis, inhibit intestinal inflammation, and improve the immune regulation of intestinal microbes [15], but the specific mechanism of action has not been reported.

Chlorogenic acid is one of the main active ingredients of many Chinese herbal medicines such as honeysuckle and eucommia [9]. Chlorogenic acid has antioxidation, antibacterial, antiviral, and antitumor effect; is blood pressure lowering, blood lipid lowering, and free radical scavenging; and improves immune regulation [10, 13, 37–40]. Studies have shown that administration of a certain amount of chlorogenic acid and caffeic acid to adult rats can significantly reduce cholesterol and tocopherol levels in tissues such as liver and plasma [41]. Studies have shown that chlorogenic acid can exert anti-inflammatory effects by inhibiting NF-*κ*B signaling pathway in lipopolysaccharide- (LPS-) induced mouse protoplasts and BV2 microglial inflammation models [42]. In recent years, the role of chlorogenic acid in the treatment of colitis has been gradually recognized. Studies have found that chlorogenic acid pretreatment can improve H_2_O_2_-induced intestinal mitochondrial damage, mitochondrial swelling, reactive oxygen species increasing, and cytochrome C release decreasing, which indicates that chlorogenic acid can reduce mitochondrial damage by improving intestinal mitochondrial ultrastructure [43]. It can also inhibit intestinal inflammation, cause weight loss, diarrhea, and colon shortening, and improve the immune regulation of intestinal microbes [44]. The results in this experiment showed that chlorogenic acid can significantly improve tissue damage, reduce inflammatory factors, and increase the expression of anti-inflammatory factors, indicating that chlorogenic acid has a protective effect on DSS-induced ulcerative colitis.

PAF can stimulate platelet and leukocyte aggregation, promote oxygen free radical production, increase adhesion molecules, lead to white blood cell adhesion, and aggregate and increase oxygen radicals [45]. PAF can dilate blood vessels, increase vascular permeability, make plasma infiltration out, reduce the blood volume, cause extensive edema of the colonic mucosa [46], stimulate intestinal secretion induced diarrhea, and promote the release of prostaglandins and leukotrienes and other inflammatory reactions [47]. PAF and platelets activate each other, making active UC abnormal blood rheology and lead to thrombotic complications; PAF binds to surface receptors of target cell and activates nuclear factor NF-*κ*B, which upregulates the synthesis and secretion of TNF-*α*, IL-1 [48, 49]. MPO is a highly expressed enzyme in neutrophils, and its expression upregulation can reflect the increase of neutrophils in tissue and then indirectly be related with inflammation. Therefore, the activity of MPO can be used to evaluate the degree of infiltration of inflammatory cells [50]. Studies have shown that MPO activity increases gradually with the increase of inflammation degree in rat model of ulcerative colitis induced by acetic acid [51].

More evidence suggests that oxidative stress and immune dysfunction caused by oxygen free radicals, genetic factors, and environmental factors are major factors in the pathogenesis of UC [52]. Studies have found that the damaging effects of intestinal oxidative stress are the main factors leading to the occurrence and/or further development of UC [53]. Therefore, effective antioxidant therapy will be one of the ways to treat UC [54, 55]. SOD is the main antioxidant substance in the body, which can effectively remove oxygen free radicals in the body and relieve oxidative damage caused by oxygen stress, thus achieving the purpose of controlling inflammation [56]. SOD has been proved to be one of the effective drugs for the treatment of UC [57]. The experimental study found that different rat UC animal models showed significant decrease in SOD content in serum and colon tissue, oxidative-antioxidant balance was broken, and the ability to scavenge oxygen freely in the body was reduced [58]. Oxidative damage caused by oxygen stress was further aggravated. After administration, the SOD related drug can effectively increase the activity of serum and colon tissue, remove excess oxygen free radicals in the body, relieve oxidative damage caused by oxygen stress, and reduce inflammation. This study found that chlorogenic acid reduced DSS-induced colonic mucosal damage, significantly prolonged the length of the colon, reduced epithelial cell degeneration / necrosis, and reduced mucosal inflammatory factors and apoptosis protein expression.

NF-*κ*B is a key transcription factor that regulates the inherent immune response and inflammation. The infiltration of immune cells in the colon and rectal mucosa is regulated by the activation of NF-*κ*B to regulate the development of the UC. The activation of NF-*κ*B is mainly achieved through the phosphorylation and dissociation of its inhibitor I*κ*B, and the phosphorylation expression of I*κ*B and NF-*κ*B p65 increased in DSS-induced colitis model [59]. We found chlorogenic acid can reduce the phosphorylation level of I*κ*B and NF-*κ*B p65 protein, thus inhibiting the activity of NF-*κ*B. MAPK/ERK/JNK pathway is the main signal transduction pathway of many factors, which is related to intestinal injury, and the three most representative pathways are ERK pathway, JNK pathway, and p38/MAPK pathway [60–63]. MAPK is a kind of highly conserved serine protease, which is involved in mediating the process of cell differentiation, proliferation, division, and apoptosis. The MAPK pathway is highly conserved, playing signal transduction function in a three-stage kinase cascade. ERK, JNK, and p38 are considered to be the main kinases in MAPK, and they can be induced by a variety of cytokines, hormones, and proteins [64]. In recent years, people have become more and more aware of the important role of MAPK/ERK/JNK signaling pathway in the pathogenesis of UC [65]. p38 is an important protein in ulcerative colitis, which can be activated by a variety of cytokines, such as hormones, IL-1, and TNF-*α*. p38 can make TATA binding protein phosphorylation in the downstream nuclear NF-*κ*B complex, regulate the transcription activity of NF-*κ*B, and then regulate the inflammatory response [66]. After ERK is activated, it can regulate the downstream targets NF-*κ*B, Bcl-2, and so on, thus affecting the inflammatory response and cell apoptosis [67]. JNK plays a regulatory role in inflammation, oxidative stress, and other processes; IL-1 can activate JNK and participate in inflammatory reactions [68]. Shi L et al. found that Beta glucan of lentinus edodes can alleviate DSS-induced colonic inflammatory cell infiltration in mice, reduce the concentration of malondialdehyde (MDA) and myeloperoxidase (MPO), and inhibit iNOS and TNF-*α*, IL-1*β*, and IL-6 and phosphorylation of JNK/ERK1/2 and p38, which suggested that Beta glucan of lentinus edodes can inhibit DSS-induced ulcerative colitis and reduce the expression of inflammatory factors, and its molecular mechanism may be involved in the inhibition of MAPK pathway and inactivate Elk-1 and activate PPAR*γ* [69]. It has been found that octreotide can stimulate NHE8 expression in colonitis mice, and somatostatin receptor 2 (SSTR2) agonist fragments and somatostatin receptor 5 (SSTR5) agonists can inhibit ERK1 / 2 phosphorylation by restoring NHE8 expression [70]. This study found that chlorogenic acid can improve the expression of ERK1/2, p-ERK, p38, p-p38, JNK, and p-JNK proteins of the MAPK/ERK/JNK pathway in DSS animal model, which suggested that chlorogenic acid has protective effect on UC and its mechanism of action may be achieved through the MAPKERK/JNK pathway.

To further validate the relationship between chlorogenic acid in the treatment of ulcerative colitis and the MAPK/ERK/JNK signaling pathway, we administered a U0126 (0.2 mg/kg) to the tail vein before administration to block MAPK/ERK/ JNK signaling pathway. The results showed that when the MAPK signaling pathway was inhibited, protective effect of chlorogenic acid on UC has been totally blocked, TNF-*α*, IL-1*β*, and IL-6 are still increased, and the anti-inflammatory factor IL-10 is increased. Antiapoptosis function, inhibition of the phosphorylation of I*κ*B, NF-*κ*B, and p65, and the regulation of MAPK/ERK/JNK signaling pathway-related protein expression disappeared. We further suggested that chlorogenic acid plays a role in the treatment of ulcerative colitis by inhibiting MAPK/ERK/JNK signaling pathway and provided a theoretical basis for chlorogenic acid to be an effective therapeutic agent for ulcerative colitis.

## Data Availability

The datasets used and/or analyzed during the current study are available from the corresponding author on reasonable request.

## References

[1] Ordás I., Eckmann L., Talamini M., Baumgart D. C., Sandborn W. J. (2012). Ulcerative colitis. *The Lancet*.

[2] Speight R. A., Mansfield J. C. (2013). Drug advances in inflammatory bowel disease. *Clinical Medicine*.

[3] Gisbert J. P., Chaparro M. (2018). Acute severe ulcerative colitis: State of the art treatment. *Best Practice & Research Clinical Gastroenterology*.

[4] Pineton de Chambrun G., Tassy B., Kollen L. (2018). The treatment of refractory ulcerative colitis. *Best Practice & Research: Clinical Gastroenterology*.

[5] Pouillon L., Baumann C., Rousseau H. (2018). Treatment persistence of infliximab versus adalimumab in ulcerative colitis: a 16-year single-center experience. *Inflammatory Bowel Diseases*.

[6] Pugliese D., Felice C., Papa A. (2017). Anti TNF-*α* therapy for ulcerative colitis: current status and prospects for the future. *Expert Review of Clinical Immunology*.

[7] Luo H., Sun Y., Li Y. (2018). Perceived stress and inappropriate coping behaviors associated with poorer quality of life and prognosis in patients with ulcerative colitis. *Journal of Psychosomatic Research*.

[8] Clifford M. N., Jaganath I. B., Ludwig I. A., Crozier A. (2017). Chlorogenic acids and the acyl-quinic acids: Discovery, biosynthesis, bioavailability and bioactivity. *Natural Product Reports*.

[9] Upadhyay R., Mohan Rao L. (2013). An outlook on chlorogenic acids-occurrence, chemistry, technology, and biological activities. *Critical Reviews in Food Science and Nutrition*.

[10] Tošović J., Marković S., Dimitrić Marković J. M., Mojović M., Milenković D. (2017). Antioxidative mechanisms in chlorogenic acid. *Food Chemistry*.

[11] Zhang L., Fan Y., Su H. (2018). Chlorogenic acid methyl ester exerts strong anti-inflammatory effects *via* inhibiting the COX-2/NLRP3/NF-*κ*B pathway. *Food & Function*.

[12] Wang H., Chu W., Ye C. (2018). Chlorogenic acid attenuates virulence factors and pathogenicity of Pseudomonas aeruginosa by regulating quorum sensing. *Applied Microbiology and Biotechnology*.

[13] Kim H., Pan J. H., Kim S. H., Lee J. H., Park J.-W. (2018). Chlorogenic acid ameliorates alcohol-induced liver injuries through scavenging reactive oxygen species. *Biochimie*.

[14] Kang T. Y., Yang H. R., Zhang J. (2013). The studies of chlorogenic acid antitumor mechanism by gene chip detection: the immune pathway gene expression. *Journal of Analytical Methods in Chemistry*.

[15] Zhang Z., Wu X., Cao S. (2017). Chlorogenic acid ameliorates experimental colitis by promoting growth of Akkermansia in mice. *Nutrients*.

[16] Rtibi K., Grami D., Wannes D. (2018). Ficus carica aqueous extract alleviates delayed gastric emptying and recovers ulcerative colitis-enhanced acute functional gastrointestinal disorders in rats. *Journal of Ethnopharmacology*.

[17] Sun Y., Liu W. Z., Liu T., Feng X., Yang N., Zhou H. F. (2015). Signaling pathway of MAPK/ERK in cell proliferation, differentiation, migration, senescence and apoptosis. *Journal of Receptors and Signal Transduction*.

[18] Rousseau S., Martel G. (2016). Gain-of-function mutations in the toll-like receptor pathway: TPL2-mediated ERK1/ERK2 MAPK activation, a path to tumorigenesis in lymphoid neoplasms?. *Frontiers in Cell and Developmental Biology*.

[19] Liu F., Yang X., Geng M., Huang M. (2018). Targeting ERK, an achilles’ heel of the MAPK pathway, in cancer therapy. *Acta Pharmaceutica Sinica B (APSB)*.

[20] Sui X., Kong N., Ye L. (2014). P38 and JNK MAPK pathways control the balance of apoptosis and autophagy in response to chemotherapeutic agents. *Cancer Letters*.

[21] Setia S., Nehru B., Sanyal S. N. (2014). Upregulation of MAPK/Erk and PI3K/Akt pathways in ulcerative colitis-associated colon cancer. *Biomedicine & Pharmacotherapy*.

[22] Shi L., Lin Q., Li X. (2017). Alliin, a garlic organosulfur compound, ameliorates gut inflammation through MAPK-NF-*κ*B/AP-1/STAT-1 inactivation and PPAR-*γ* activation. *Molecular Nutrition & Food Research*.

[23] Sroufe I. A., Gardner T., Bresnahan K. A., Quarnberg S. M., Wiedmeier P. R. (2017). Insights into the pathophysiology of ulcerative colitis: interleukin-13 modulates STAT6 and p38 MAPK activity in the colon epithelial sodium channel. *The Journal of Physiology*.

[24] Ji L., Jiang P., Lu B., Sheng Y., Wang X., Wang Z. (2013). Chlorogenic acid, a dietary polyphenol, protects acetaminophen-induced liver injury and its mechanism. *The Journal of Nutritional Biochemistry*.

[25] Zhang W.-F., Yang Y., Su X. (2016). Deoxyschizandrin suppresses dss-induced ulcerative colitis in mice. *Saudi Journal of Gastroenterology*.

[26] Zhang H., Zhang Z., Song G. (2017). Development of an XBP1 agonist, HLJ2, as a potential therapeutic agent for ulcerative colitis. *European Journal of Pharmaceutical Sciences*.

[27] Da Silva B. C., Lyra A. C., Rocha R., Santana G. O. (2014). Epidemiology, demographic characteristics and prognostic predictors of ulcerative colitis. *World Journal of Gastroenterology*.

[28] Kwak M. S., Cha J. M., Lee H. H. (2018). Emerging trends of inflammatory bowel disease in South Korea: a nationwide population‐based study. *Journal of Gastroenterology and Hepatology*.

[29] Crowley E., Muise A. (2018). Inflammatory bowel disease: what very early onset disease teaches us. *Gastroenterology Clinics of North America*.

[30] Weisshof R., El Jurdi K., Zmeter N., Rubin D. T. (2018). Emerging therapies for inflammatory bowel disease. *Advances in Therapy*.

[31] Luo S., Wen R., Wang Q. (2018). Rhubarb peony decoction ameliorates ulcerative colitis in mice by regulating gut microbiota to restoring Th17/Treg balance. *Journal of Ethnopharmacology*.

[32] Qi Q., Liu Y.-N., Jin X.-M. (2018). Moxibustion treatment modulates the gut microbiota and immune function in a dextran sulphate sodium-induced colitis rat model. *World Journal of Gastroenterology*.

[33] Wang Y., Tang Q., Duan P., Yang L. (2018). Curcumin as a therapeutic agent for blocking NF-*κ*B activation in ulcerative colitis. *Immunopharmacology and Immunotoxicology*.

[34] Zhang D., Ren Y.-B., Wei K. (2018). Herb-partitioned moxibustion alleviates colon injuries in ulcerative colitis rats. *World Journal of Gastroenterology*.

[35] Xu L., Yang Z.-L., Li P., Zhou Y.-Q. (2009). Modulating effect of Hesperidin on experimental murine colitis induced by dextran sulfate sodium. *Phytomedicine*.

[36] Pandurangan A. K., Ismail S., Saadatdoust Z., Esa N. M. (2015). Allicin alleviates dextran sodium sulfate- (DSS-) induced ulcerative colitis in BALB/c mice. *Oxidative Medicine and Cellular Longevity*.

[37] Lee B., Lee D. G. (2018). Depletion of reactive oxygen species induced by chlorogenic acid triggers apoptosis-like death in Escherichia coli. *Free Radical Research*.

[38] Shao P., Zhang J. F., Chen X. X., Sun P. L. (2015). Microwave-assisted extraction and purification of chlorogenic acid from by-products of Eucommia Ulmoides Oliver and its potential anti-tumor activity. *Journal of Food Science and Technology*.

[39] Watanabe T., Arai Y., Mitsui Y. (2006). The blood pressure-lowering effect and safety of chlorogenic acid from green coffee bean extract in essential hypertension. *Clinical and Experimental Hypertension*.

[40] Wan C., Wong C. N., Pin W. (2013). Chlorogenic acid exhibits cholesterol lowering and fatty liver attenuating properties by up-regulating the gene expression of PPAR-*α* in hypercholesterolemic rats induced with a high-cholesterol diet. *Phytotherapy Research*.

[41] Frank J., Kamal-Eldin A., Razdan A., Lundh T., Vessby B. (2003). The dietary hydroxycinnamate caffeic acid and its conjugate chlorogenic acid increase vitamin E and cholesterol concentrations in Sprague-Dawley rats. *Journal of Agricultural and Food Chemistry*.

[42] Hwang S. J., Kim Y.-W., Park Y., Lee H.-J., Kim K.-W. (2014). Anti-inflammatory effects of chlorogenic acid in lipopolysaccharide- stimulated RAW 264.7 cells. *Inflammation Research*.

[43] Zhou Y., Zhou L., Ruan Z. (2016). Chlorogenic acid ameliorates intestinal mitochondrial injury by increasing antioxidant effects and activity of respiratory complexes. *Bioscience, Biotechnology, and Biochemistry*.

[44] Chen J., Xie H., Chen D. (2018). Chlorogenic acid improves intestinal development via suppressing mucosa inflammation and cell apoptosis in weaned pigs. *ACS Omega*.

[45] Palur Ramakrishnan A. V. K., Varghese T. P., Vanapalli S., Nair N. K., Mingate M. D. (2017). Platelet activating factor: a potential biomarker in acute coronary syndrome?. *Cardiovascular Therapeutics*.

[46] Pałgan K., Bartuzi Z. (2015). Platelet activating factor in allergies. *International Journal of Immunopathology and Pharmacology*.

[47] Peplow P. V., Mikhailidis D. P. (1990). Platelet-activating factor (PAF) and its relation to prostaglandins, leukotrienes and other aspects of arachidonate metabolism. *Prostaglandins, Leukotrienes and Essential Fatty Acids*.

[48] Poubelle P. E., Gingras D., Demers C. (1991). Platelet-activating factor (PAF-acether) enhances the concomitant production of tumour necrosis factor-alpha and interleukin-1 by subsets of human monocytes. *The Journal of Immunology*.

[49] Kim K., Cho K., Jang K. Y. (2014). Platelet-activating factor enhances tumour metastasis via the reactive oxygen species-dependent protein kinase casein kinase 2-mediated nuclear factor- *κ* B activation. *The Journal of Immunology*.

[50] Gu P., Zhu L., Liu Y., Zhang L., Liu J., Shen H. (2017). Protective effects of paeoniflorin on TNBS-induced ulcerative colitis through inhibiting NF-kappaB pathway and apoptosis in mice. *International Immunopharmacology*.

[51] Ghasemi-Pirbaluti M., Motaghi E., Najafi A., Hosseini M. J. (2017). The effect of theophylline on acetic acid induced ulcerative colitis in rats. *Biomedicine & Pharmacotherapy*.

[52] Adams S. M., Bornemann P. H. (2013). Ulcerative colitis. *American Family Physician*.

[53] Jin X., Chen D., Zheng R.-H., Zhang H., Chen Y.-P., Zun X. (2017). MiRNA-133a-UCP2 pathway regulates inflammatory bowel disease progress by influencing inflammation, oxidative stress and energy metabolism. *World Journal of Gastroenterology*.

[54] Boeing T., de Souza P., Bonomini T. J. (2018). Antioxidant and anti-inflammatory effect of plumieride in dextran sulfate sodium-induced colitis in mice. *Biomedicine & Pharmacotherapy*.

[55] Kim K. J., Park J. M., Lee J. S. (2018). Oligonol prevented the relapse of dextran sulfate sodium-ulcerative colitis through enhancing NRF2-mediated antioxidative defense mechanism. *Journal of Physiology and Pharmacology*.

[56] Younus H. (2018). Therapeutic potentials of superoxide dismutase. *International Journal of Health Sciences (Qassim)*.

[57] Peppercorn M. A. (1990). Advances in drug therapy for inflammatory bowel disease. *Annals of Internal Medicine*.

[58] Sakthivel K. M., Guruvayoorappan C. (2013). Amentoflavone inhibits iNOS, COX-2 expression and modulates cytokine profile, NF-*κ*B signal transduction pathways in rats with ulcerative colitis. *International Immunopharmacology*.

[59] Gao W., Zhang L., Wang X., Yu L., Wang C., Gong Y. (2018). The combination of indirubin and isatin attenuates dextran sodium sulfate induced ulcerative colitis in mice. *The International Journal of Biochemistry & Cell Biology*.

[60] Ming Y., Chao H., Chu S., Luo C. (2018). Heparin-binding epidermal growth factor-like growth factor (HB-EGF) protected intestinal ischemia-reperfusion injury through JNK and p38/MAPK-dependent pathway for anti-apoptosis. *Pediatrics and Neonatology*.

[61] Ran X., Li Y., Chen G. (2018). Farrerol ameliorates TNBS-induced colonic inflammation by inhibiting ERK1/2, JNK1/2, and NF-*κ*B signaling pathway. *International Journal of Molecular Sciences*.

[62] Muta Y., Matsuda M., Imajo M. (2018). Dynamic ERK signaling regulation in intestinal tumorigenesis. *Molecular & Cellular Oncology*.

[63] Wang X., Cui X., Zhu C. (2018). FKBP11 protects intestinal epithelial cells against inflammationinduced apoptosis via the JNKcaspase pathway in Crohn's disease. *Molecular Medicine Reports*.

[64] Chang L., Karin M. (2001). Mammalian MAP kinase signalling cascades. *Nature*.

[65] Gao Z., Yu C., Liang H. (2018). Andrographolide derivative CX-10 ameliorates dextran sulphate sodium-induced ulcerative colitis in mice: involvement of NF-*κ*B and MAPK signalling pathways. *International Immunopharmacology*.

[66] Kim B.-W., More S. V., Yun Y.-S. (2016). A novel synthetic compound MCAP suppresses LPS-induced murine microglial activation in vitro via inhibiting NF-kB and p38 MAPK pathways. *Acta Pharmacologica Sinica*.

[67] Lee K.-C., Chen W.-T., Liu Y.-C., Lin S.-S., Hsu F.-T. (2018). Amentoflavone inhibits hepatocellular carcinoma progression through blockage of ERK/NF-*κ*B activation. *In Vivo*.

[68] Panahi G., Pasalar P., Zare M., Rizzuto R., Meshkani R. (2018). High glucose induces inflammatory responses in HepG2 cells via the oxidative stress-mediated activation of NF-*κ*B, and MAPK pathways in HepG2 cells. *Archives of Physiology and Biochemistry*.

[69] Shi L., Lin Q., Yang T. (2016). Oral administration of: Lentinus edodes *β*-glucans ameliorates DSS-induced ulcerative colitis in mice via MAPK-Elk-1 and MAPK-PPAR*γ* pathways. *Food & Function*.

[70] Li X., Cai L., Xu H. (2016). Somatostatin regulates NHE8 protein expression via the ERK1/2 MAPK pathway in DSS-induced colitis mice. *American Journal of Physiology-Gastrointestinal and Liver Physiology*.

